# A Pleasant Remedy for Toothache

**Published:** 1880-01

**Authors:** T. C. Osborn

**Affiliations:** Greensboro, Ala.


### A PLEASANT REMEDY FOR TOOTHACHE.

BY T. C. OSBORN, M. D., GREENSBORO, ALA.

Our cook presented herself to me with a swollen cheek, asking for something to relieve the toothache, from which she had been suffering all night, and for which she refused to have the tooth extracted. As there was nothing of the usual kind at hand, I was on the point of telling her to call later at my office, or go to a dentist, when it occurred to my mind that there was in the house a vial of *compound tincture of benzoin*, which I had been using upon a young mother as a protection against sore nipples.

After cleansing the decayed tooth I saturated a pledget of cotton lint with the tincture, and packed it well into the cavity, hoping this would suffice for the time, and bidding her come back in two or three hours if she was not relieved. I was turning away when she remarked that it might not be necessary, perhaps, as the pain was already gone. Supposing her faith had a large share in the relief, I would not allow myself to think that the medicine had anything to do with the cure any more than so much hot water would have done.

But when I arrived at my office there were two other patients awaiting me with the same affliction, and I determined, by way of experiment, to use the same remedy. To my agreeable surprise both patients declared themselves immediately relieved, and begged a vial of the tincture for future use.

During the winter a number of similar cases applied, and were instantly relieved by the same treatment, all expressing much satisfaction with the remedy.

In December I told my druggist of the discovery, and recommended him to sell it to any person applying for "toothache drops." This, he reports, he has done, and that every one seems delighted with the medicine. * * *

---

MR. HEPBURN, in a paper read before the Odontological Society of London, says "that the direct action of nicotine on the teeth is decidedly beneficial. The alkalinity of the smoke must necessarily neutralize any acid secretion which may be present in the oral cavity, and the antiseptic property of the nicotine tends to arrest putrefactive changes in carious cavities."—*Dental Register.*

